# Tartaric acid – Determination of L-(+)- and D-(–)-tartaric acid in workplace air using ion chromatography (IC): Air Monitoring Method – Translation of the German version from 2025

**DOI:** 10.34865/am52683e10_4or

**Published:** 2025-12-22

**Authors:** Ulrich Prott, Claus-Peter Maschmeier, Ralph Hebisch, Uta Lewin-Kretzschmar, Andrea Hartwig, MAK Commission

**Affiliations:** 1 Federal Institute for Occupational Safety and Health (BAuA) Friedrich-Henkel-Weg 1–25 44149 Dortmund Germany; 2 Federal State Saxony-Anhalt Gebrüder-Bethmann-Str. 18 06862 Dessau-Roßlau Germany; 3 German Social Accident Insurance, Institution for the raw materials and chemical industry, Prevention - Department of Hazardous Substances, Biological Agents and Analytical Chemistry Kurfürsten-Anlage 62 69115 Heidelberg Germany; 4 Institute of Applied Biosciences. Department of Food Chemistry and Toxicology. Karlsruhe Institute of Technology (KIT) Adenauerring 20a, Building 50.41 76131 Karlsruhe Germany; 5 Permanent Senate Commission for the Investigation of Health Hazards of Chemical Compounds in the Work Area. Deutsche Forschungsgemeinschaft, Kennedyallee 40, 53175 Bonn, Germany. Further information: Permanent Senate Commission for the Investigation of Health Hazards of Chemical Compounds in the Work Area | DFG

**Keywords:** tartaric acid, air analyses, analytical method, workplace measurement, hazardous substance, ion chromatography, conductivity detection, IC, glass fibre filter, liquid desorption, Luft, Luft, Luft, Luft, air, air, air, air

## Abstract

The working group “Air Analyses” of the German Senate Commission for the Investigation of Health Hazards of Chemical Compounds in the Work Area (MAK Commission) developed and verified the presented analytical method. It is used to determine the levels of L-(+)-tartaric acid [87-69-4] and D-(–)-tartaric acid [147-71-7] (occurring as inhalable particles) individually or as a racemic mixture [133-37-9] that occur in the workplace air. The method covers concentrations in the range from one tenth up to twice the current Occupational Exposure Limit Value (OELV) of 2 mg/m^3^ (inhalable fraction). The method is also suitable for measuring the short-term exposure limit (STEL; excursion factor 2) for the inhalable fraction. Samples are collected by drawing a defined volume of air through a glass fibre filter, which is inserted in a GSP sampling system, using a flow regulated pump at a volumetric flow rate of 3.5 l/min. Exposure during the shift is measured with a sampling period of 2 hours and the short-term exposure with a period of 15 minutes. Tartaric acid deposited on the glass fibre filter is extracted with the IC eluent and analysed by ion chromatography using conductivity detection. The quantitative determination is based on multiple-point calibrations with external standards. A relative limit of quantification (LOQ) of 0.00043 mg/m^3^ is obtained for an air sample volume of 420 litres. As the LOQ for a sample volume of 52,5 litres is 0.0034 mg/m^3^, the STEL can also be measured. The recovery is 100–104% and the expanded uncertainty is 19–21% for a 2-hour sampling and 20–21% for a 15-minute sampling.

**Table:** 

**Method number**	1
**Application**	Air analysis
**Analytical principle**	Ion chromatography with conductivity detection (IC)

## 1 Characteristics of the method

**Table:** 

**Precision:**	Standard deviation (rel.):	*s* = 0.67–0.94%
	Expanded uncertainty:	*U* = 19–21%
	in the range of 0.1–2 mg/m^3^ and for n = 6 determinations	
**Limit of quantification:**	0.018 mg/l in the measurement solution	
	0.00043 mg/m^3^ for an air sample volume of 0.42 m^3^ and a sampling period of 2 hours 0.0034 mg/m³ for an air sample volume of 0.0525 m³ and a sampling period of 15 minutes	
**Recovery:**	*η* = 100–104%	
**Sampling recommendations:**	Sampling period:	2 h
	Air sample volume:	420 l
	Volumetric flow rate:	3.5 l/min
	For short-term measurements:	15 min; 52.5 l

## 2 Description of the substance

### Tartaric acid

**Fig. 1:**
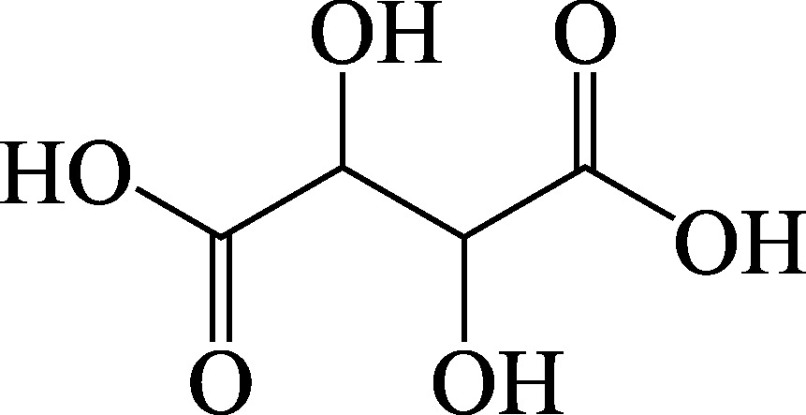
Structural formula of tartaric acid (without stereoisomers)

Tartaric acid [526-83-0] (see Figure 1, also called 2,3-dihydroxybutanedioic acid, 2,3-dihydroxysuccinic acid, racemic tartaric acid, threaric acid, E334) has three stereoisomers. The isomers D-(–)-tartaric acid [147-71-7] and *meso*-tartaric acid [147-73-9] and the racemic mixture of L-(+)- and D-(–)-tartaric acid [133-37-9], which is called racemic tartaric acid, rarely occur in nature. With few exceptions, L-(+)-tartaric acid [87-69-4] is the only isomer that forms naturally. The salts of tartaric acid are known as tartrates.

Tartaric acid is a white, odourless crystalline solid that has a sour taste. It is readily soluble in water and a strong acid.

The L-form of tartaric acid is found in many plants and fruits. It may occur in free form or as a potassium, calcium, or magnesium salt. Grape juice contains both free tartaric acid and potassium bitartrate. In winemaking, potassium bitartrate precipitates out together with calcium tartrate during fermentation, forming crystals that are known as “wine diamonds”. D-(–)-tartaric acid rarely occurs in nature, but is found, for example, in the leaves of the West African *Bauhinia reticulata* tree. Tartaric acid forms complexes with heavy metal ions such as copper, iron and lead (e.g. Fehling’s solution for determining urinary glucose levels) (RÖMPP-Redaktion and Hartmann-Schleier 2007).

Tartaric acid has been approved as a food additive (E334) and serves as a flavour enhancer and as an acidulant, e.g. in ice cream, lemonade and baking powder (Hartwig 2015; RÖMPP-Redaktion and Hartmann-Schleier 2007). It is employed in such applications as electroplating, glass silvering and metal dyeing, for the production of antimony potassium tartrate and plasticizers, as an auxiliary in the manufacture of lacquers and as an intermediate in the production of other chemicals. Additionally, the substance is used as an acid and reducing agent in dyeing and printing processes in the textile industry (RÖMPP-Redaktion and Hartmann-Schleier 2007).

An occupational exposure limit value (OELV) of 2 mg/m^3^ was established for L-(+)-tartaric acid [87-69-4]. The substance is classified in Peak Limitation Category I with an excursion factor of 2 (AGS 2025). The same value as the OELV has been listed in the List of MAK and BAT Values as the MAK value of L-(+)-tartaric acid (DFG 2024). The substance has been classified in the same peak limitation category with the same excursion factor. The substance data for tartaric acid are given in Table 1. Neither an OELV nor a MAK value has been established for D-(–)-tartaric acid and *meso*-tartaric acid.

**Tab. 1:** Substance data for tartaric acid (Huisman et al. 2013; RÖMPP-Redaktion and Hartmann-Schleier 2007)

Name	L-(+)-tartaric acid	D-(–)-tartaric acid	DL-(±)-tartaric acid
CAS No.	87-69-4	147-71-7	133-37-9
Molar mass [g/mol]	150.09	150.09	150.09
Physical state at 20 °C	solid	solid	solid
Density at 20 °C [g/cm^3^]	1.76	1.76	1.76
Vapour pressure at 25 °C [Pa]^a)^	–	–	1.4 × 10^–9^–3.2 × 10^–1^
Melting point [°C]	169–170	169–170	205–206
Boiling point at 1013 hPa [°C]	–	–	–
Solubility in water [g/l] at 20 °C	1390	1390	1390
Criteria of assessment			
Germany: OELV, MAK value (AGS 2025; DFG 2024)	2 mg/m^3^	–	–
Peak limitation category (excursion factor) (AGS 2025; DFG 2024)	I(2)	–	–

^a)^ Ten values are given in Huisman et. al. (2013) for the vapour pressure of tartaric acid; of these, eight values are smaller or equal to 1.8 × 10^–4^ Pa. The values for the vapour pressure were determined in experiments using solid and liquid tartaric acid as well as calculated using suitable models.

## 3 General principles

The method was developed using a racemate (DL-(±)-tartaric acid) and may therefore be used to determine concentrations of L-(+)- and D-(–)-tartaric acid. For the sake of clarity, the racemic mixture DL-(±)-tartaric acid is referred to only as tartaric acid in the following. As *meso*-tartaric acid hardly occurs in nature, it has no relevance as a hazardous substance at the workplace and was therefore not taken into consideration for the development of the method. Due to its physical properties, it elutes much earlier than DL-(±)-tartaric acid on the column chosen for this method (cf. Section 10.8).

This analytical method is used to determine L-(+)- and D-(–)-tartaric acid in the workplace air in the concentration range from 0.1 to 2 times the MAK value and the currently valid OELV for L-(+)-tartaric acid of 2 mg/m^3^ (AGS 2025; DFG 2024). The method is also suitable for determining the short-term concentration with an excursion factor of 2 (AGS 2025; DFG 2024; DIN 2021).

Samples are taken by drawing a defined volume of air from the breathing zone through a GSP sampling head containing a glass fibre filter using a sampling pump. After elution and ion chromatographic separation, a conductivity detector is used to detect the acid anion (tartrate). The quantitative analysis of tartaric acid is based on two multiple point calibrations with external calibration.

## 4 Equipment, chemicals and solutions

### 4.1 Equipment

For sampling:

- Pump for personal sampling, volumetric flow rate of 3.5 l/min (e.g. GilAirplus, Sensidyne, Saint Petersburg, FL, USA, supplied by e.g. DEHA Haan & Wittmer GmbH, 71296 Heimsheim, Germany)
- Personal sampling system for hazardous substances (PGP) with a GSP sampling head for the inhalable dust fraction and an intake cone for a flow rate of 3.5 l/min and a suitable filter cassette (e.g. from DEHA Haan & Wittmer GmbH, 71296 Heimsheim, Germany)
- Mass flow meter for 0–20 l/min (e.g. TSI 4146, from TSI GmbH, 52068 Aachen, Germany)
- Glass fibre filter, binder-free, diameter 37 mm (e.g. MN85/90 BF, Ref: 4060037, from Macherey-Nagel GmbH & Co. KG, 52355 Düren, Germany)

For sample preparation and the analytical determination:

- Analytical balance (e.g. XPE205 Delta Range, from Mettler-Toledo GmbH, 35396 Gießen, Germany)
- Stainless steel spatula
- Weighing boats made of glass
- Volumetric flasks, 10 ml, 25 ml, 100 ml, 250 ml and 2000 ml (e.g. from Brand GmbH + Co. KG, 97877 Wertheim, Germany)
- Variable piston pipettes with volumes of 10–100 µl and 100–1000 µl (e.g. Reference 2, from Eppendorf AG, 22366 Hamburg, Germany)
- Bulb pipette, 10 ml (e.g. from Brand GmbH + Co. KG, 97877 Wertheim, Germany) with pipette controller
- Stainless steel tweezers
- Amber glass bottles, 10 ml with Teflon seals (e.g. G18 septum, CS-Chromatographie Service GmbH, 52379 Langerwehe, Germany)
- Dispenser, 10 ml (e.g. Dispensette, from Brand GmbH + Co. KG, 97877 Wertheim, Germany)
- Laboratory shaker (e.g. KS 15 A Control shaker, from Edmund Bühler GmbH, 72379 Hechingen, Germany)
- Disposable syringes made of polyethylene (PE), 10 ml (e.g. BD Discardit II, from Becton Dickinson and Company, Franklin Lakes, NJ, USA)
- Disposable cannulas, 1.2 × 40 mm (e.g. BD Microlance 3, from Becton Dickinson and Company, Franklin Lakes, NJ, USA)
- Disposable filters made of PTFE with Luer tip, diameter 25 mm, pore width 0.45 µm (e.g. Chromafil Xtra H-PTFE-45/25, Ref: 729246, from Macherey-Nagel GmbH & Co. KG, 52355 Düren, Germany)
- Autosampler vials made of PE, 2.5 ml with perforated stoppers (e.g. Art No. 6.2743.040 and Art No. 6.2743.077, from Metrohm Deutschland GmbH & Co. KG, 70794 Filderstadt, Germany)
- Ion chromatograph with degasser, column oven, autosampler, chemical and CO_2_ suppression as well as conductivity and UV detector (e.g. 930 Compact IC Flex, from Metrohm Deutschland GmbH & Co. KG, 70794 Filderstadt, Germany)
- Metrosep A Supp 16-250/4.0 separation column with Metrosep A Supp 16 Guard/4.0 pre-column (e.g. Art No. 6.1031.430 and Art No. 6.1031.500, from Metrohm Deutschland GmbH & Co. KG, 70794 Filderstadt, Germany)

### 4.2 Chemicals

- Tartaric acid, anhydrous, ≥ 99.5% (e.g. from Carl Roth GmbH + Co. KG, 76185 Karlsruhe, Germany)
- Sodium carbonate, anhydrous, p.a., ≥ 99.9% (e.g. Art No. 1.06392.1000, from Merck KGaA, 64271 Darmstadt, Germany)
- Sodium bicarbonate, p.a., ≥ 99.5% (e.g. Art No. 1.06329.1000, from Merck KGaA, 64271 Darmstadt, Germany)
- Sulfuric acid, 2.5 mol/l (5 N) in aqueous solution (e.g. AVS TITRINORM volumetric solution, for suppressor regeneration, Art No. 30138293, from VWR International, 94126 Fontenay-sous-Bois, France)
- Ultrapure water (ρ ≥ 18.2 MΩ × cm at 25 °C)

### 4.3 Solutions

The following solutions were prepared using the chemicals listed in Section 4.2:

**Eluent stock solution:** (1.1 mol sodium carbonate/l and 0.5 mol sodium bicarbonate/l in ultrapure water)

11.6589 g of sodium carbonate (anhydrous) and 4.2005 g of sodium bicarbonate are weighed into a 100-ml volumetric flask. The flask is then filled to the mark with ultrapure water and shaken.

**Eluent** (IC eluent and for the elution of the glass fibre filters): (5.5 mmol sodium carbonate/l and 2.5 mmol sodium bicarbonate/l in ultrapure water)

A bulb pipette is used to add 10 ml of the eluent stock solution to a 2000-ml volumetric flask containing about 500 ml of ultrapure water. The flask is then filled to the mark with ultrapure water and shaken.

**Tartaric acid stock solution 1** (for calibration): (40 mmol/l in ultrapure water)

603.38 mg of tartaric acid (99.5% purity) is weighed out precisely into a 100-ml volumetric flask. The flask is then filled to the mark with ultrapure water and shaken. The tartaric acid stock solution 1 has a concentration of 6.0036 g/l or 40 mmol/l.

**Tartaric acid stock solution 2** (for the control standards): (20 mmol/l in ultrapure water)

301.69 mg of tartaric acid (99.5% purity) is weighed out precisely into a 100-ml volumetric flask. The flask is then filled to the mark with ultrapure water and shaken. The tartaric acid stock solution 2 has a concentration of 3.0018 g/l or 20 mmol/l.

The stock solutions can be used for at least 4 weeks if stored at room temperature and protected from light.

### 4.4 Calibration and control standards

**Calibration standards:**

Tartaric acid stock solution 1 is used to prepare 12 calibration solutions as follows:

For the lower concentration range, tartaric acid stock solution 1 is added to five 10-ml volumetric flasks, each containing about 5 ml of eluent, in the volumes listed in Table 2. Similarly, for the upper concentration range, tartaric acid stock solution 1 is added to seven 25-ml volumetric flasks in the volumes given in Table 3. The volumetric flasks are filled to the mark with eluent and then shaken. The concentrations of the calibration standards are given in Table 2 and 3.

**Tab. 2:** Pipetting scheme for the preparation of the five calibration standards for tartaric acid in the lower concentration range

**Calibration standard**	**Tartaric acid stock solution 1** **[µl/10 ml]**	**Concentration** **[mg/l]**
I	10	6.004
II	30	18.01
III	50	30.02
IV	70	42.03
V	90	54.03

**Tab. 3:** Pipetting scheme for the preparation of the seven calibration standards for tartaric acid in the upper concentration range

**Calibration standard**	**Tartaric acid stock solution 1** **[µl/25 ml]**	**Concentration** **[mg/l]**
VI	200	48.03
VII	300	72.04
VIII	400	96.06
IX	500	120.1
X	600	144.1
XI	700	168.1
XII	800	192.1

The calibration standards cover about 7 to 230% of the OELV at an air sample volume of 0.42 m^3^ and an eluate volume of 10 ml.

**Control standards:**

About 5 ml of eluent are filled into 10-ml and 25-ml volumetric flasks. 400 µl or 250 µl of tartaric acid stock solution 2, respectively, is added to the flasks as given in Table 4. The volumetric flasks are then filled to the mark with eluent and shaken. The concentrations of the control standards are listed in Table 4. Control standard I is in the middle of the lower calibration range and control standard II in the middle of the upper calibration range.

**Tab. 4:** Pipetting scheme for the preparation of the control standards

**Control standard**	**Tartaric acid stock solution 2** **[µl]**	**Final volume** **[ml]**	**Concentration** **[mg/l]**
I	250	25	30.02
II	400	10	120.1

## 5 Sampling and sample preparation

### 5.1 Preparation of the sample carriers

The glass fibre filters do not need to be prepared in a specific way. The sample carriers are prepared by first placing a supporting sieve into a filter cassette and then placing a glass fibre filter on the sieve. The caps of the filter cassette remain closed with the lids provided until sampling begins.

The batch of glass fibre filters used has to be checked for possible blank values.

### 5.2 Sampling

For sampling, the filter cassette equipped with a supporting sieve and a glass fibre filter is placed into the GSP sampling head (Möhlmann 2025) and fitted with an intake cone for a flow rate of 3.5 l/min. Sampling is carried out over a period of 15 minutes to determine the short-term concentration and for 2 hours to determine the shift average, resulting in air sample volumes of 52.5 litres and 420 litres, respectively. Sampling can be carried out as stationary or personal sampling. After sampling, the flow rate must be tested for constancy. The sample should be discarded if the deviation from the adjusted flow rate is greater than ± 5% (DIN 2023). The filter cassette with the loaded filter is sealed with caps and the cassette is transported to the laboratory.

Each series of samples must include a field blank. The only difference in the handling of this sample and the analytical samples is that an air sample is not drawn through the filter. The field blank is stored and processed in the same manner as the samples.

### 5.3 Sample preparation

No later than 24 hours after sampling, tweezers are used to carefully remove the filter from the filter cassette and to transfer it into a 10-ml amber glass bottle. The filter is then covered with 10 ml of IC eluent using a dispenser and the bottle is briefly shaken by hand. As the pH of the elution solution is in the alkaline range, tartaric acid is present in the form of the acid anion (tartrate), which is the analyte that is determined chromatographically. The sealed bottle is stored in the refrigerator at 2 to 8 °C until analysis.

To prepare the samples, the amber glass bottles with the loaded filters are first removed from the refrigerator and allowed to return to room temperature. The samples are shaken in a laboratory shaker for 60 minutes at 300 revolutions per minute.

A disposable syringe is used to remove about 4 ml of the liquid from the suspension. The first half of a millilitre that passes through the disposable filter (0.45 µm pore width) is discarded. The filtrate that follows is collected in an autosampler vial. The amber bottle with the remaining liquid is re-sealed and returned to the refrigerator as a reserve sample.

The field blank is prepared and analysed in the same manner as the collected samples.

The additional preparation and analysis of a lab blank is recommended.

## 6 Operating conditions

**Table:** 

**Apparatus:**	Ion chromatograph with degasser, column oven, autosampler, chemical and CO_2_ suppression
**Pre-column:**	MetroSep A Supp 16 Guard/4.0
**Separation column:**	MetroSep A Supp 16-250/4.0
**Column temperature:**	50 °C
**Detector:**	conductivity detector
**Mobile phase:**	5.5 mmol sodium carbonate and 2.5 mmol sodium bicarbonate, isocratic
**Flow rate:**	0.75 ml/min
**Injection volume:**	10 µl
**Run time:**	40 min

Tartrate has a retention time of about 24.5 minutes under the given conditions.

## 7 Analytical determination

The analytical determination is performed by injecting 10 µl of each of the samples prepared as described in Section 5.3 into the ion chromatograph and analysing the samples under the conditions specified in Section 6. The analysis is based on the calibration curve for the low or high concentration range depending on the tartaric acid concentration in the sample. If the determined concentrations lie above the calibrated range, suitable dilutions are prepared with the eluent and then analysed. The field and lab blanks are analysed in the same manner as the analytical samples.

## 8 Calibration

External calibration:

The calibration standards from Section 4.4 are analysed as described in Sections 6 and 7 to derive the calibration functions. The resulting peak areas are plotted against the respective concentrations.

The calibration functions are linear in the investigated concentration range and should be checked every working day as part of the routine analysis. For this purpose, each analytical series must include a control standard with a known concentration for analysis.

A re-calibration must be performed if the analytical conditions change or the quality control indicates that this is necessary.

## 9 Calculation of the analytical result

Equation 1 is used to calculate the concentration of tartaric acid in the workplace air taking into consideration the air sample volume, the eluate volume, the dilution and the recovery. Equation 1 does not require correction if a recovery of 100 ± 5% is obtained for the range of one tenth to twice the limit value.



(1)

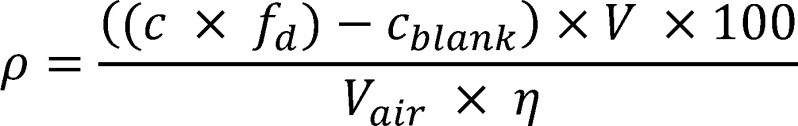

rho equal to startFraction open parenthesis open parenthesis c times f subscript d close parenthesis minus c subscript blank close parenthesis times V times 100 divided by V subscript air times etha endFraction.



where:

**Table:** 

*ρ*	is the mass concentration of tartaric acid in the air sample in mg/m^3^
*c*	is the concentration of tartaric acid in the measurement solution in mg/l
*f_d_*	is the dilution factor
*c_blank_*	is the concentration of the field blank (mean) in mg/l
*V*	is the volume of the eluate in litres (in this case 0.01 litres)
*V_air_*	is the air sample volume in m^3^ (calculated using the volumetric flow rate and the sampling period, in this case 0.42 m^3^ after a sampling period of 2 hours)
*ƞ*	is the recovery in %

## 10 Reliability of the method

The characteristics of the method were determined according to DIN EN 482 (DIN 2021) and DIN 32645 (DIN 2008). The method was fully validated.

### 10.1 Repeatability

The repeatability was evaluated by analysing one calibration standard in the medium concentration range of each calibration curve every day over a period of 6 days. A relative standard deviation of 0.94% was determined for the medium range of the lower calibration curve (30.18 mg/l) and a value of 0.67% for the medium range of the upper calibration curve (121.7 mg/l).

### 10.2 Recovery and reproducibility

To determine the recovery, a tartaric acid solution was used to spike sets of six filters with three different concentrations of tartaric acid.

Spiking solution 1 was prepared by weighing 4218 mg of tartaric acid (99.5% purity) into a 250-ml volumetric flask, filling the flask to the mark with ultrapure water and then shaking the flask by hand. Spiking solution 1 had a concentration of 16 790 mg/l.

Spiking solutions 2 and 3 were prepared by diluting spiking solution 1 in the volumes given in Table 5 with ultrapure water to obtain a final volume of 100 ml. The table gives also the concentrations of the spiking solutions. The dilution factors apply to spiking solution 1.

**Tab. 5:** Preparation of spiking solutions 2 and 3 for the purpose of spiking the filters

**Spiking solution**	**Dilution factor**	**Volume of spiking solution 1** **[ml]**	**Volume** **[ml]**	**Concentration** **[mg/l]**
2	2	50	100	8394
3	20	5	100	839.4

The filters were loaded with 100 µl of spiking solutions 1 to 3 according to Table 6; the concentrations are thus equivalent to one tenth of the OELV, the OELV and twice the OELV at an air sample volume of 0.42 m^3^.

**Tab. 6:** Pipetting scheme for preparing the spiked filters and the recovery

**Concentration equivalent to^a)^**	**Spiking solution**	**Volume of the spiking solution** **[µl]**	**Mass per filter** **[mg]**	**Recovery ** **[%]**	**Standard deviation (rel.)** **[%]**
0.1 OELV	3	100	0.08395	103.8	0.63
1 OELV	2	100	0.8395	100.5	2.89
2 OELV	1	100	1.679	100.1	0.57

^a)^ at an eluate volume of 10 ml and an air sample volume of 0.42 m^3^

After drying, 0.42 m^3^ of laboratory air was drawn through the spiked filters at a volumetric flow rate of 3.5 l/min, a room temperature of 20 °C and 50% relative humidity. The caps of the filter cassettes were then closed and the filters were prepared the following day as described in Section 5.3.

The analytical determination was performed as described in Sections 6 and 7. Unlike the concentrations equivalent to 0.1 OELV, the concentrations equivalent to 1 OELV and 2 OELV were calculated based on the calibration curve in the upper concentration range.

The recovery for all three concentrations is given in Table 6.

The mean recovery for tartaric acid was 101.5%.

### 10.3 Capacity of the sample carrier

The experiments carried out with three filters spiked with concentrations equivalent to 1 OELV and an air sample volume of 0.84 m^3^ – which corresponds to a sampling period of 4 hours at a volumetric flow rate of 3.5 l/min – obtained the same values for recovery with a mean recovery of 99.9%.

The recovery does not need to be taken into consideration when calculating the results for a sampling period lasting 4 hours and a concentration in air equivalent to the OELV.

### 10.4 Expanded uncertainty for the entire method

The expanded uncertainty was determined by spiking sets of six glass fibre filters with different masses of tartaric acid according to Section 10.2, letting the filters dry and then performing all steps of sample preparation and analysis as described in Sections 5.3, 6 and 7. As a result, the filters were spiked with concentrations equivalent to one tenth of the OELV, the OELV and twice the OELV at an air sample volume of 0.42 m^3^.

The expanded uncertainty was estimated taking into consideration all relevant influencing parameters according to DIN EN 482 (DIN 2021) and DIN EN ISO 21832 (DIN 2020) and calculated using the Excel tool provided by the IFA ([Bibr ref_4KHV8EG5]) for the calculation of expanded uncertainty.

The combined, concentration-dependent uncertainties were calculated by combining the contributions from all sources of uncertainty. The concentration-dependent expanded uncertainty for the entire method was calculated by multiplying the values with the probability factor k = 2 (for a 95% confidence level).

Table 7 provides an overview of all relevant sources of uncertainty, distinguishing between low, medium and high filter loads. The low, medium and high concentrations are equivalent to one tenth of the OELV, the OELV and twice the OELV, respectively. The uncertainties were calculated for sampling periods of 15 minutes and 120 minutes.

**Tab. 7:** Determination of sources of uncertainty; data in %

**Uncertainty**	**Sampling period of 15 minutes**	**Sampling period of 120 minutes**
*u* sampling, transport, storage	9.3	8.9
*u* recovery low concentration	4.0	4.0
*u* recovery medium concentration	1.6	1.6
*u* recovery high concentration	1.5	1.5
*u* analytical variability	3.4	3.5
*U* expanded low concentration	21.4	20.8
*U* expanded medium concentration	20.1	19.4
*U* expanded high concentration	20.1	19.4

The analytical method has an expanded uncertainty of 19 to 21% for the measurement range from one tenth to twice the limit value and a sampling period of 2 hours. The expanded uncertainty was between 20 and 21% for the measurement range from half to twice the short-term concentration and a sampling period of 15 minutes.

### 10.5 Influence of humidity

The influence of humidity was additionally investigated at a relative humidity of 20% and 80% and an air temperature of 21 °C. In this range, the recovery of tartaric acid was not dependent on the relative humidity.

The relative humidity does not need to be taken into consideration when calculating the analytical results if it lies between 20 and 80% and the temperature at sampling is about 21 °C. The analytical procedure must be re-evaluated if the testing conditions are different from those described.

### 10.6 Limit of quantification

The limit of quantification was determined according to DIN 32645 (DIN 2008) based on an equidistant 10-point calibration in the lower concentration range. The absolute limit of quantification was 0.018 mg/l.

The 10-point calibration was prepared as follows with a serial dilution of a measured amount of tartaric acid:

600.98 mg of tartaric acid was weighed into a 100-ml volumetric flask, the flask was filled to the mark with ultrapure water and then shaken. The tartaric acid concentration was 5980 mg/l (solution 1) after taking the purity into consideration.

750 µl of solution 1 was added to a 250-ml volumetric flask containing about 50 ml of eluent. The flask was then filled to the mark with eluent and shaken. This tartaric acid solution had a concentration of 17.94 mg/l (solution 2).

Ten 25-ml volumetric flasks were each filled with 5 ml of eluent. Solution 2 was added in the volumes given in Table 8. The flasks were then filled to the mark with eluent and shaken. The concentrations of the 10 calibration standards are listed in Table 8.

**Tab. 8:** Pipetting scheme for the preparation of the solutions for the 10 calibration standards used to determine the limit of quantification

**Calibration standard**	**Volume of solution 2** **[µl]**	**Final volume** **[ml]**	**Tartaric acid concentration** **[mg/l]**
I	100	25	0.0718
II	140	25	0.1005
III	180	25	0.1292
IV	220	25	0.1579
V	260	25	0.1866
VI	300	25	0.2153
VII	340	25	0.2440
VIII	380	25	0.2727
IX	420	25	0.3014
X	460	25	0.3301

The limit of quantification for tartaric acid was 0.018 mg/l at a 95% confidence interval. This is equivalent to a relative limit of quantification of 0.00043 mg/m^3^ (shift average) after taking into consideration an eluate volume of 10 ml and an air sample volume of 0.42 m^3^. At an air sample volume of 0.0525 m^3^, the respective value is 0.0034 mg/m^3^ (short-term concentration).

### 10.7 Storage stability

Using a procedure similar to that described in Section 10.2 for the recovery, sets of 15 filters were spiked with concentrations equivalent to 0.1 OELV, 1 OELV and 2 OELV as listed in Table 6.

After the filters were air-dried, 0.42 m^3^ of clean air was drawn through the filters at a volumetric flow rate of 3.5 l/min. The filter cassettes were sealed with caps. On the following day, the filters were removed with tweezers and transferred into 10-ml amber glass bottles. A dispenser was used to cover the filters with 10 ml of IC eluent. The amber glass bottles were closed, shaken briefly by hand and then stored in the refrigerator at about + 5 °C. On the day of analysis, the bottles were removed from the refrigerator, allowed to return to room temperature and then prepared and analysed according to Sections 5.3, 6 and 7.

Sets of 3 filters per concentration were prepared as described in Section 5.3 either on the day of spiking (not stored in the refrigerator) or after 1 week, 2 weeks, 3 weeks or 4 weeks and then analysed the same day.

Storage periods of 1, 2 or 3 weeks did not have a noticeable effect on the recovery. The values for the recovery after storage in the refrigerator for 4 weeks are given in Table 9.

**Tab. 9:** Recovery determined after storage in the refrigerator for 4 weeks at about 5 °C

**Tartaric acid concentration per filter** **[mg]**	**Equivalent to OELV^a)^**	**Recovery** **[%]**
0.08395	0.1	94.3
0.8395	1	97.6
1.679	2	99.3

^a)^ at an eluate volume of 10 ml and an air sample volume of 0.42 m^3^

The mean recovery was 97.1% after storage in the refrigerator for 4 weeks. Glass fibre filters that are spiked and eluted the day after spiking have a storage stability of 4 weeks when stored in the refrigerator at about 5 °C.

### 10.8 Selectivity

Tartaric acid has three stereoisomers. The method can be used to determine the concentrations of L-(+)- and D-(–)-tartaric acid. The two isomers have identical retention times. Their signals are also identical at the same concentration. The method was developed using a racemic isomer (DL-(±)-tartaric acid).

The analytical procedure was not validated for *meso*-tartaric acid [147-73-9] because its retention time is considerably lower under the same analytical conditions and the compound is not relevant as a hazardous substance at the workplace.

The method cannot be used to distinguish between tartaric acid and the tartrates (e.g. alkali salts). The tartrates, which are soluble in the eluent, are detected and reported as tartaric acid. Table 10 provides a list of common tartrates and the values for their solubility in water. These tartrates are sufficiently soluble in water to evaluate the concentrations in air in the range of the OELV of tartaric acid.

**Tab. 10:** Water solubility of a few select tartrates (IFA 2025 a, b, c, d, e)

**Substance**	**CAS No.**	**Water solubility at 20 °C** **[g/l]**	**After reaching the saturation concentration, equivalent to a concentration in air of^a)^** **[mg/m³]**
Ammonium tartrate	3164-29-2	428	8300
Dipotassium tartrate	921-53-9	235.3	3700
Antimony potassium tartrate	6535-15-5	55	290
Potassium bitartrate	868-14-4	6.2	120
Potassium sodium tartrate	304-59-6	630	11 000

^a)^ at an eluate volume of 10 ml and an air sample volume of 0.42 m^3^, calculated as tartaric acid

**Fig. 2:**
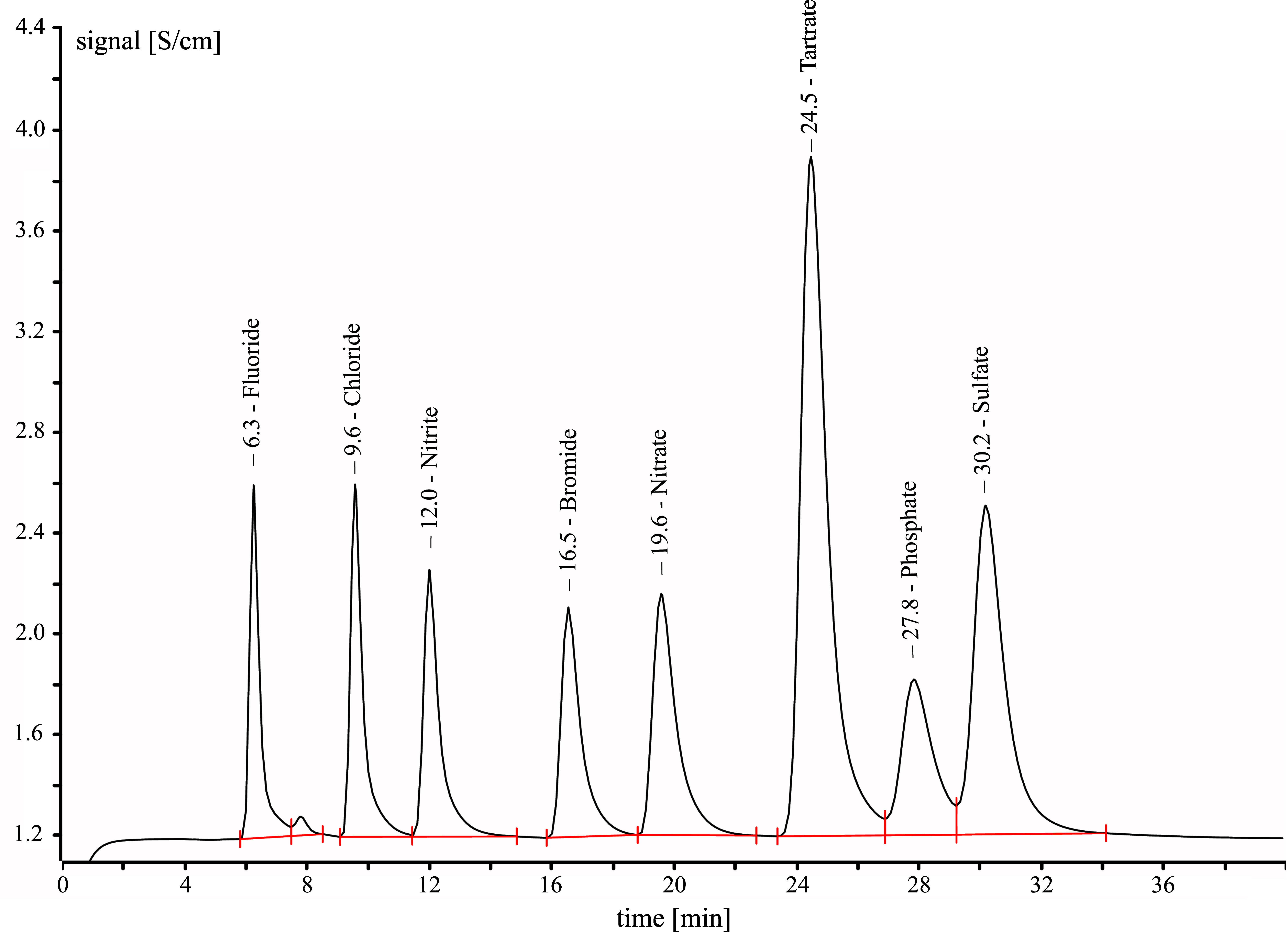
Ion chromatogram of tartaric acid (tartrate, 24.5 min) and other anions: fluoride (6.3 min), chloride (9.6 min), nitrite (12.0 min), bromide (16.5 min), nitrate (19.6 min), phosphate (27.8 min), sulfate (30.2 min). Tartaric acid concentration 6 mg/l

The anions fluoride, bromide, chloride, nitrate, nitrite and sulfate and the anions of the dicarboxylic acids oxalic acid and adipic acid do not interfere with the analysis (see Figure 2).

If present, succinic acid produces a false positive signal because tartrate and succinate co-elute. They cannot be separated under the chromatographic conditions chosen for this method.

A complete baseline separation of the signals of phosphate and the anion of glutaric acid (glutarate), which co-elute, and the tartrate signal was not achieved. However, this did not noticeably interfere with the analysis of tartaric acid.

The anion of malic acid (malate) elutes shortly before and the anion of malonic acid (malonate) shortly after tartrate. As a result, a baseline separation was not achieved for these acids either. In both cases, the signal returns to the baseline by 70% if tested at the same concentration as tartaric acid. A test using a solution containing equal concentrations of malic acid and tartaric acid yielded about 10% more tartaric acid and a test using a solution containing equal concentrations of tartaric acid and malonic acid yielded about 10% less tartaric acid.

Similar to the dicarboxylic acids mentioned above, tartaric acid is visibly active on the UV detector at a wavelength of 210 nm and can be determined in the calibrated range. The limit of quantification for tartaric acid is about 2.6 mg/l at a measured wavelength of 210 nm and a 95% confidence interval. After taking into consideration an eluate volume of 10 ml and an air sample volume of 0.42 m^3^, this is equivalent to a relative limit of quantification of 0.062 mg/m^3^ (shift average). Absorption on the UV detector can be used as an additional qualitative identifier for tartaric acid, e.g. to separate tartaric acid from phosphate, which is not UV-active.

Field blanks prepared at the same time as the samples were used to test for blank values. None were detected. No blank values were detected on the glass fibre filters or in the eluent.

## 11 Discussion

The method is suitable for determining L-(+)- and D-(–)-tartaric acid in the workplace air in the concentration range from one tenth to twice the currently valid OELV of 2 mg/m^3^. The method is also suitable for monitoring compliance with the short-term concentration. Temperatures up to 40 °C are not expected to affect the results because of the low vapour pressure of tartaric acid (cf. Table 1).

The method can additionally be used to determine soluble tartrates that occur in the workplace air in particle form. These are detected as tartaric acid.
